# Sonar Objective Detection Based on Dilated Separable Densely Connected CNNs and Quantum-Behaved PSO Algorithm

**DOI:** 10.1155/2021/6235319

**Published:** 2021-01-18

**Authors:** Zhen Wang, Buhong Wang, Jianxin Guo, Shanwen Zhang

**Affiliations:** ^1^School of Information and Navigation, Air Force Engineering University, Xi'an 710077, China; ^2^School of Information Engineering, Xijing University, Xi'an 710123, China

## Abstract

Underwater sonar objective detection plays an important role in the field of ocean exploration. In order to solve the problem of sonar objective detection under the complex environment, a sonar objective detection method is proposed based on dilated separable densely connected convolutional neural networks (DS-CNNs) and quantum-behaved particle swarm optimization (QPSO) algorithm. Firstly, the dilated separable convolution kernel is proposed to extend the local receptive field and enhance the feature extraction ability of the convolution layers. Secondly, based on the linear interpolation algorithm, a multisampling pooling (MS-pooling) operation is proposed to reduce the feature information loss and restore image resolution. At last, with contraction-expansion factor and difference variance in the traditional particle swarm optimization algorithm introduced, the QPSO algorithm is employed to optimize the weight parameters of the network model. The proposed method is validated on the sonar image dataset and is compared with other existing methods. Using DS-CNNs to detect different kinds of sonar objectives, the experiments shows that the detection accuracy of DS-CNNs reaches 96.98% and DS-CNNs have better detection effect and stronger robustness.

## 1. Introduction

**Figure 1:**
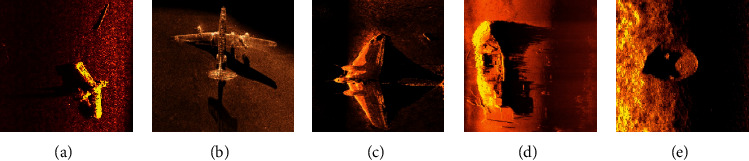
Original sonar images.

Ocean is an essential part of the Earth's life support system and is a huge natural treasure house with rich mineral and biological resources [1]. With the development of the world economy, the requirement in the field of national defense and national economy, such as marine resource development and dam safety detection, makes underwater imaging detection technology receive more and more attention [2]. Underwater imaging technology includes optical imaging and sonar imaging. The optical imaging effect is relatively close, and the imaging effect is unfavorable in the muddy water area [3]. Sonar imaging has the advantages of remote action and strong penetrating ability. It is especially suitable for muddy water area, so it is widely used in marine detection, underwater objective detection, and dam defect detection [4]. Due to the chaotic water medium of the underwater acoustic channel and the long transmission of the acoustic wave itself, the images acquired by the imaging sonar often have the problems of huge noise, severe distortion, blurred target edge, and low resolution, which seriously affect the underwater objective detection and identification [5]. Some sonar images are shown in Figure 1.

Since the limitations of underwater sonar images, many optical image processing methods must be modified to apply to underwater acoustic image processing. Many researches have proposed a series of methods for the detection of sonar objective. The combination of feature extraction algorithm and classifiers has been widely used in sonar objective detection. For example, Lianantonakis and Petillot [6] proposed an unsupervised active contour model for sonar image segmentation. In this method, by extracting the texture features of the side-scan sonar image and extracting the weight parameters of different texture features through the curve evolution equation, the weight parameters were used to construct the active contour model. Elias et al. [7] proposed a sonar target detection method based on image texture features and independent correlation analysis. This method uses a traditional feature extraction method to extract multiple texture features of sonar images and then uses principal component analysis to perform dimensional reduction on texture features. The sonar objective is detected using independent correlation analysis. Markov is a statistical image segmentation algorithm with advantages of less model parameters and strong spatial constraints [8]. Mignotte et al. [9] used a new iterative condition estimation method to detect the objective region of the sonar image. The method establishes the Markov model by using the mixed distribution theorem and the marker parameter estimation and then uses the maximum likelihood estimation and the least square method to calculate the Markov model parameters. And the sonar image is segmented using the established Markov model. Cacciola et al. [10] used the finite element analysis (FEA) design and Misfit minimization methods to detect abnormal objective data. Collet et al. [11] proposed a hierarchical Markov random field model to solve the problem of sonar target detection. By combining maximum likelihood estimation and least squares method, they proposed a proportional casual multigrid algorithm, and the traditional Markov is improved as a hierarchical model for the label field of the pyramid structure, which is capable of accurately segmenting submarine shadows and seafloor reverberation noise. Yan et al. [12] proposed a sonar objective detection method based on extreme learning machine. This method assigns multiple extracted features by the convolutional neural network as central pixel to one of the categories and then, based on the central pixel, detects the objective area of the sonar image. The snake model uses some control points that form a certain shape as a template and through the elastic deformation of the template itself, matches the local features of the image to achieve harmony, and completes the energy function minimization to achieve image segmentation [13]. Angiulli et al. [14] used the second-order parabolic equations based on mathematical statistics to model and detect abnormal objective. Peng and Guo [15] proposed an improved snake model to detect the sonar objective. The snake model is defined as an energy minimizing spline which is influenced by external constraint force, and it can guide the image forces to pull toward feature. Compared with the traditional snake model, this improved snake model greedy algorithm can converge to the contours more quickly and more stably. In order to solve the problem of sonar target detection, Wang et al. [16] proposed a target detection algorithm based on the neutrosophic set and diffusion maps. Firstly, the neutrosophic set image of the sonar image is obtained, and then, the single gray threshold algorithm is used to extract the diffusion map of the sonar image; finally, the target region is detected using the improved target score equation. The combination of particle swarm optimization (PSO) algorithm and clustering algorithm has been widely used in the field of sonar image segmentation. In order to solve the problem of low efficiency and precision of existing sonar image segmentation methods, Zhao et al. [17] proposed a sonar image segmentation method based on PSO and neutrosophic set. The method first constructs image grayscale in NS domain. The symbiotic matrix expresses the fine texture features of the image, and it is based on the two-dimensional maximum entropy principle. And then, it is optimized by the particle swarm optimization algorithm to obtain the optimal two-dimensional segmentation threshold vector to achieve accurate segmentation of the objective region of the sonar image. Guo et al. [18] used a multibit particle system to encode the particles and proposed a sonar objective detection algorithm based on heuristic particle swarm optimization and fuzzy clustering. It calculates the particle swarm adaptive variance based on its own optimal particles and global optimal particles and updates the particle position in real time using a multibit quantum revolving gate. The output of the improved PSO algorithm is used to initialize the K-means clustering center. Based on the idea of fuzzy membership matrix in FCM and combined with the isolated spatial information features of noise, the objective region of sonar image is segmented. All the above methods use the traditional image processing method to detect the sonar objective. Although some effects are obtained, there are many problems with these methods, such as complex algorithm structure and poor robustness.

In recently years, deep learning has been widely used in the field of image processing. Since it can extract the deep semantic features of images and does not need to manually set the classifier, deep learning has many advantages in image segmentation, detection, and recognition [19–21]. Many researchers have applied deep learning to sonar image processing to detect sonar objective. Song et al. [22] constructed deep convolutional neural networks (CNNs) to segment the sonar image. In the training process, the model uses multiple convolution kernels of the same size as the input image to extract features. Then, the Markov model is used to optimize the results obtained by CNNs, and the trained network model is used to segment different types of sonar images. Valdenegro [23] proposed a sonar objective detection algorithm based on a convolution neural network. This method detects the underwater small objective area by the constructed deep network model and proposes the regional suggestion network to generate a suggestion box for the objective region. Finally, the threshold optimization algorithm is used to optimize the parameters of the network model. The results show that the method can complete the detection of multiple small targets detection. Zhu et al. [24] first use the convolution neural network to extract the deep semantic features of the sonar image and then train the SVM classifier based on the manually labeled data. In the process of objective detection, the target region is detected using the sliding window with the kernel. The experiment results indicate that its performance is better than the objective detection algorithm of histogram of oriented gradients (HOGs) and local binary pattern (LBP). Based on the CNNs, Dzieciuch et al. [25] proposed a sonar objective detection method based on a combination of nonlinear classifier and CNNs. This method can extract abstract and complex features of sonar images, and it can accurately detect the objective region with complex background environments. Since the method introduces nonlinear operations, it has a faster training speed and higher detection accuracy. As there are few sonar images that can be used for training, Chen and Summers [26] proposed an unsupervised CNNs model for sonar objective detection. The method constructs a semisupervised ladder network and uses it for a small sample sonar image to execute classification and positioning. The semisupervised semantic segmentation and objective localization of sonar images are realized by pixel-level classification of the entire sonar image and by using the ladder network to realize semantic segmentation and objective localization of sonar images. Since noise affects the segmentation accuracy of sonar images, Jiao et al. [27] proposed a sonar image segmentation method based on improved fully convolution neural networks (FCNs). This method improves the original mean square error loss function by using cosine distance. The improved method can complete the accurate segmentation of the submarine sonar image. Williams and Dugelay [28] proposed a method for multiview sonar image classification based on image fusion and deep learning methods. The method can accurately classify different types of sonar images and complete the classification task for sonar images containing noise and complex background. Wang and Zhang [29] proposed a method based on multiscale multicolumn convolution neural network (MSMC-CNNs) for sonar objective detection. The parallel network structure design of MSMC-CNNs can enhance the multiscale feature extraction ability, and experimental result shows it can complete the detection task for different sonar objectives. Bin et al. [30] proposed a sonar objective detection method based on transfer learning and geometric feature constraints. The method first uses transfer learning to improve the detection accuracy of the network model and then employs the geometric feature constraint method to improve the detection efficiency of the model. Finally, different kinds of sonar images are detected with the constructed model.

The evolutionary computing (EC) and swarm intelligence (SI) are mature global optimization methods with high robustness and wide applicability, which have the characteristics of self-organization, self-adaptation, and self-learning [31]. Moreover, the EC and SI algorithms are not limited by the nature of the problem and can effectively deal with complex optimization problems that are difficult to solve by traditional optimization algorithm. Based on the advantages of EC and SI, many researches used it to solve the weight parameter optimization problem of CNNs. Alejandro et al. [32] proposed the EvoDeep evolutionary algorithm to optimize the structure and weight parameters of CNNs, which can improve the classification accuracy and maintain a valid layers sequence. Watanabe and Shinozaki [33] used genetic algorithm (GA) and CMA-ES algorithm to optimize the weight parameters of CNNs. In this method, the GA is used to represent CNN structure as directed acyclic graphs and optimized the graph structure by CMA-ES algorithm. Real et al. [34] proposed a new neural network evolution method to evolve the structure of CNNs. This method can effectively optimize the weight parameters of CNNs, which achieve high classification accuracy on the CIFAR-10 and CIFAR-100 datasets. Chung and Shin [35] proposed a GA-optimized multichannel CNN method for stock market prediction. The method uses the GA algorithm to optimize the CNN parameters, and the experimental results show that GA algorithm can effectively improve the compute efficiency and prediction accuracy. To solve the problem of CNN optimization, Połap et al. [36] used a multithreading mechanism for weight parameters selection, in which the multicore mechanism is used to select the best weight among all parallel training networks. Moayedi et al. [37] optimized the artificial neural network (ANN) by the combination of dragonfly algorithm (DA), whale optimization algorithm (WOA), and invasive weed optimization (IWO). Experimental results show that this method can effectively improve the prediction accuracy of the ANN model. The above methods show that the use of EC and SI algorithms can effectively optimize the structure and weight parameters of CNNs.

At present, the sonar objective detection method based on deep learning has better effect than the traditional image processing method. However, due to the complicated structure of the model and the long training time of the weight parameter, the existing models are less practical. In order to solve the problems of deep learning method in sonar objective detection, this paper is based on U-Net, dilated convolution, separable convolution, and DenseNet to propose dilated separable densely connected convolution neural networks (DS-CNNs) for sonar objective detection. The main contributions of this paper are listed as follows:

1. The dilated separable convolution kernel is proposed to expand the local receptive field of the traditional convolution kernel and enhance the feature extraction ability of the convolution layers.
2. Based on the linear interpolation algorithm, a multisampling pooling method is proposed to solve the problem of feature and sample information loss caused by convolution operation.
3. The quantum-behaved particle swarm optimization (QPSO) algorithm is proposed to replace the traditional backpropagation (BP) algorithm to train the weight parameters of the network model.
4. The DS-CNN is validated and compared with the state-of-the-art methods on the sonar image dataset, and the robustness of DS-CNNs is analyzed.

The rest of this paper is organized as follows. In Section 2, the related work is introduced, including U-Net, dilated convolution, separable convolutions, and DenseNet. In Section 3, DS-CNNs are described in detail, including model parameters, multisampling pooling, and QPSO algorithm. The experiments on the sonar image dataset are implemented in Section 4, and the conclusion is given in Section 5.

## 2. Related Works

In this section, the related works are introduced, which includes U-Net, separable convolution, dilated convolution, and DenseNet.

### 2.1. U-Net Network Model

**Figure 2:**
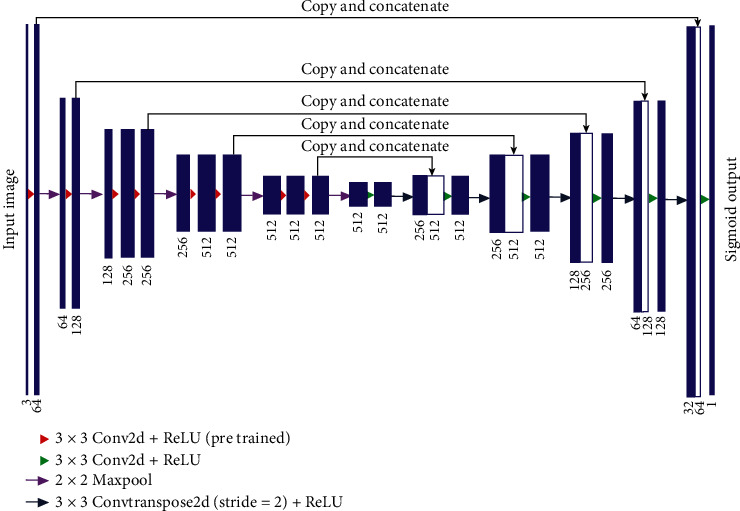
Structure of U-Net network model.

The structure of U-Net [38] is the same as that of FCN [39] and SegNet [40], which includes two parts of mutually symmetric contraction network and expansion network. The contraction network part in U-Net includes convolutional layers, pooling layers, and activation function, whose main function is to complete feature extraction of the input image. The expanded network portion includes deconvolution and upsampling and is mainly used to restore the size resolution of the feature map. Moreover, the uses of skip connections and pooling indexes in the U-Net structure can better improve the segmentation accuracy. The U-Net structure is shown in Figure 2. It can be seen from Figure 2 that the U-Net contraction network and the expansion network form a U-shaped structure. The convolution and pooling operations of the contraction network can extract the multilayer semantic information of the image, while the expansion network realizes the restoration of the image features through multilayer deconvolution and upsampling operations, and the expanded network is restored by the pooled index operation, so the boundary information of the feature map is continuously restored during the process.

U-Net is characterized by the mutual mapping of the contraction network and the expansion network. In the expansion network, the image feature map in the contraction network is merged and the missing boundary information is complemented, thereby improving the classification accuracy of the pixels. Moreover, U-Net's U-shaped structure makes the model more intuitive for the cutting and splicing process of feature maps. By splicing high-level semantic features with low-level semantic features, the model can obtain more context information and detailed information. The contraction network in U-Net consists of ten convolutional layers and four max-pooling layers. Each two convolutional layers and one max-pooling layer form one downsampling block, and there are five downsampling block structures in the contraction network. Each downsampling block is composed of two 3 × 3 sized convolution layers and one 2 × 2 sized max-pooling layer. After each downsampling block operation, the size of the output feature map turns out to be one-half of the input feature images, and the number of channels becomes twice of the original. The extension network and the contraction network correspond to each other, and they are mainly used for image upsampling operations. They include five upsampling blocks of the same structure, each of which contains two 3 × 3 convolution layers and one 2 × 2 max-pooling layer. The deconvolution layer uses a nonlinear unit as the activation function. After each upsampling, the image becomes twice as large as the original feature maps, and the number of channels becomes one-half. Each pixel is classified at the end of the network using SoftMax classifier.

### 2.2. Depth-Wise Separable Convolutions

**Figure 3:**
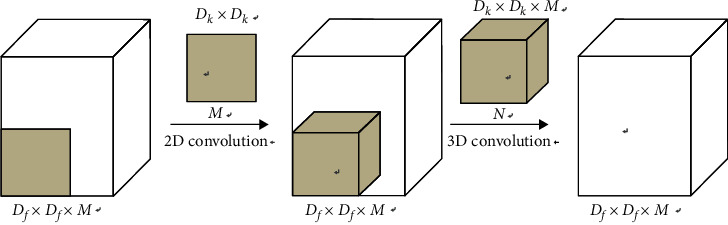
Depth-wise separable convolutions kernels.

Traditional convolutional neural networks use large-scale convolution kernels. For example, AlexNet uses an 11 × 11 size convolution kernel. Although a large local receptive field is obtained, the calculation of the parameters in the model also increases. In the GoogLeNet [41] model, multiple 3 × 3 small-scale convolution kernel cascade structures are used to reduce the parameter calculation of the model under the same conditions to maintain the original image receptive field. The small-scale convolution kernel cascade structure not only increases the depth of the network model but also enhances the nonlinear expression ability of image features. Since the traditional convolution kernel needs to convolute each channel of the input feature maps, the parameter calculation of the model is heavy. In order to solve the problem of large amount of parameter calculation, Howard et al. [42] proposed a depth-wise separable convolution kernel, which can greatly reduce the parameter calculation of the network model. The depth-wise separable convolution firstly uses two-dimensional convolution operation on each channel in the input feature map to reduce the amount of parameter calculation and then uses 1 × 1 convolution kernel to carry out three-dimensional convolution operations for the combination of different channels. The structure of the depth-wise separable convolution is shown in Figure 3. Assume that the size of the input feature maps is *D*_*f*_ × *D*_*f*_, the number of channels is *M*, and the size of the convolution kernel is *D*_*M*_ × *D*_*M*_, which contain *N* convolution kernels.

### 2.3. Dilated Convolutions

**Figure 4:**
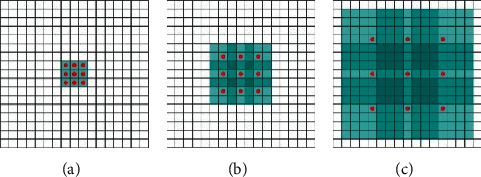
Dilated convolution kernel structures with three dilation rate parameters: (a) coefficient is 1; (b) coefficient is 2; (c) coefficient is 3.

The use of standard convolution kernels in convolutional layers can lead to insufficient information extraction capabilities in the process of feature extraction of images. Moreover, when the feature reduction is performed using the pooling operation, although the parameter calculation amount of the network model is reduced, a large amount of useful information also get lost. The use of dilated convolution can effectively avoid the information loss caused by the pooling operation, and it can also expand the local receptive field of the convolution kernel [43]. Moreover, the dilated convolution does not need to compress the resolution of the image and retains a large amount of internal space information in the image, which has better effect in image segmentation than conventional convolution kernel. The dilated convolution contains the dilated coefficient to control the size. The larger the dilated coefficient is, the larger the receptive field corresponding to the convolution kernel of the same calculation amount is. As shown in Figure 4, the dilated convolution kernels of different dilated coefficients are, respectively, indicated, where the red dot area indicates the position where the convolution operation is required, and the blue area indicates the local receptive field of the dilated convolution. It can be seen from Figure 4 that the dilated convolution kernel gradually increases with the increase in the dilated coefficient.

### 2.4. DenseNet

**Figure 5:**
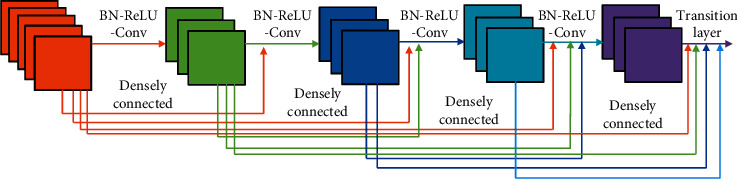
Densely connected structure.

In order to enhance the feature extraction ability of convolutional neural networks, Huang et al. [44] proposed the densely connected convolutional networks (DenseNet) structure based on ResNet [45]. DenseNet uses a simpler feature transfer method to achieve feature reuse by connecting shallow features to deep features. The DenseNet network structure is shown in Figure 5. The output of each layer can be directly connected to the input of each layer so that the method reduces the parameters of the network model while increasing the network depth. Using densely connected structure can reduce the number of parameters, but pooling operations in the network produce feature maps of different sizes, making it impossible to connect them directly. In order to solve the problem that the feature layers of the overall structure cannot be densely connected, DenseNet employs a block design approach, using dense connections only in each block area. The output of each block will be used as the input to the next block after the pooling operation, and the densely connected operation will still be used in the next block. At the same time, a 1 × 1 convolutional layer is added to each block to reduce the thickness of the feature maps and reduce the parameter size of the network model. The overall structure of DenseNet is different from the traditional networks model. Each block of DenseNet contains a batch normalization (BN), ReLU activation function, and convolution layer. Moreover, an additional convolutional layer is added at the beginning of the DenseNet structure. This operation enables the BN and the ReLU in each block to better function on the output of the previous block. Since DenseNet uses a method that directly passes features, it is easier for the network to preserve shallow features, which helps to produce accurate segmentation results.

## 3. Proposed Method

In this section, the proposed dilated separable densely connected convolutional neural network (DS-CNN) structure, quantum-behaved particle swarm optimization (QPSO) algorithm, and multisampling pooling (MS-pooling) are introduced.

### 3.1. DS-CNN Model

**Figure 6:**
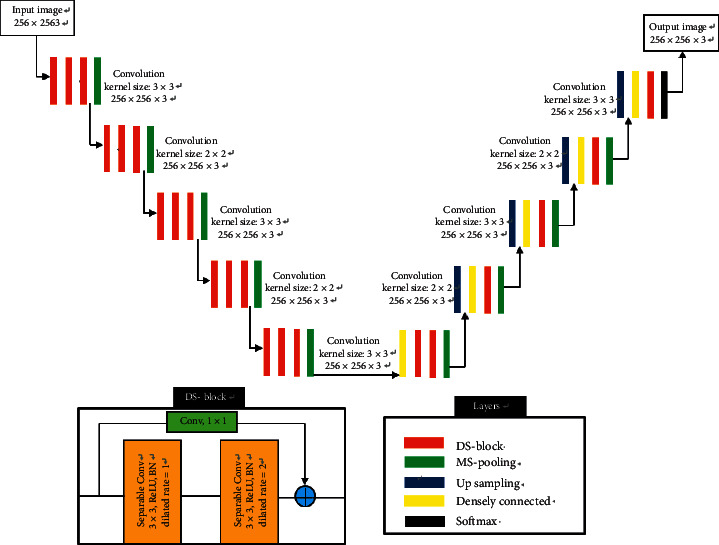
The architecture of DS-CNNs.

Based on U-Net [38], dilated convolution [41], depth-wise separable convolution [42], and DenseNet [44], a dilated separable densely connected convolutional neural networks (DS-CNNs) for sonar objective detection is proposed. DS-CNNs not only improve the detection accuracy of sonar objective but also reduce training and segmentation time. The structure of DS-CNNs is shown in Figure 6. DS-CNNs include encoder network structure and decoder network structure, where the input of the encoder network is the original sonar image and the encoder network is composed of five dilated separable convolution blocks. Each convolution block contains two dilated separable convolution layers. Each dilated separable convolution layer consists of depth-wise separable convolution of 3 × 3 and 1 × 1 sizes, with different dilated coefficients set in these depth-wise separable convolutions. Convolution kernel with a dilated coefficient of 1 is used to extract the detailed features of the image, and the convolution kernel with a dilated coefficient of 2 can extract the edge features of the image. The convolution combination of different dilated coefficients can capture the multiscale context information of the image. In order to avoid the gradient explosion problem caused by the increase in network depth and also to make better use of the feature information between different layers, the input and output of the dilated separable convolution blocks are connected by using densely connection and 1 × 1 convolution kernel. Moreover, the different dilated separable convolutional blocks in the encoder network are connected using the MS-pooling layers, and the MS-pooling size is 2 × 2. As the number of network layers increases, the encoder network's ability to characterize images also becomes stronger. Each time the convolution kernel is pooling, the channel number of the convolution kernel is doubled. The specific parameters are shown in Table 1.

The decoder network structure performs upsampling on the feature map extracted in the encoder network and hierarchically maps the high-level semantic features extracted in the encoder network to a semantic segmentation map of the same size. The decoder network structure includes three dilated separable convolution blocks, and different convolution blocks are connected by bilinear interpolation sampling layer operation. In the upsampling process, the traditional fully convolutional neural networks only upsample the final mapping result to obtain an image of the same size as the input. However, this upsampling operation ignores the shallow output information, and this information also has some characterization capabilities for the image. Therefore, in order to better keep the spatial information of the image, DS-CNNs fuse the shallow layers information with the corresponding decoder network structure through densely connection (DC) so that the shallow context information can be propagated to a higher resolution. And the deep abstract features are combined with shallow representation features to locate image grayscale category information.

### 3.2. Multisampling Pooling

The purpose of the pooling operation in the convolutional neural network is to reduce the dimensionality of the convolutional feature map and selectively compress the feature map to further extract the convolutional features. The pooling options in CNNs include mean-pooling and max-pooling, wherein the mean-pooling is to obtain the average value of the convolution feature maps, and the max-pooling is to obtain the maximum value of the convolution feature maps. The calculation formula for mean-pooling and max-pooling is as follows:

(1) $$\begin{array}{c} {\text{out\_put}}^{k}=\frac{1}{m\times m}\overset{m}{\underset{i=1}{\sum }}\overset{m}{\underset{j=1}{\sum }}{\text{in\_put}}_{ij}^{k}, \end{array}$$

where *m* represents the size of the pooling area, in_put_*ij*_^*k*^ represents the *k* − th pooling area, and out_put^*k*^ represents the pooling feature value corresponding to the *k* − th pooling area.

(2) $$\begin{array}{c} {\text{out\_put}}^{k}=\underset{i,j\in \left[1,m\right]}{\max}\left({\text{in\_put}}_{ij}^{k}\right), \end{array}$$

where max(·) represents the maximum function, and the returned result is the maximum value in the in_put_*ij*_^*k*^ region.

In the process of using the mean-pooling and max-pooling, the mean-pooling is to use the mean value in the pooling area as the characteristic response, which is easy to cause the deviation of the estimated mean value in the feature map, resulting in the blurring effect. The max-pooling is to obtain the characteristic response by obtaining the maximum value in the pooling area, so it is easy to ignore the detailed features extracted in each pooling layer. In order to solve the problems of the traditional pooling operation, multisampling pooling (MS-pooling) method is proposed instead of the original pooling operation. MS-pooling is proposed based on the interpolation algorithm, so it is necessary to insert a basis function to fit the data in MS-pooling. The corresponding value of the function *f*(·) at the pixel point *p*(*x*, *y*) is obtained by weighted average of the sixteen points closest thereto, and MS-pooling uses two polynomial interpolation cubic functions of the sampling points in the rectangular grid, among which the basic functions are as follows:

(3) $$\begin{array}{c} S\left(w\right)=\left\{\begin{array}{cc} 1-2{\left|w\right|}^{2}+{\left|w\right|}^{3}, & \left|w\right|<1,\\ 4-8\left|w\right|+5{\left|w\right|}^{2}-{\left|w\right|}^{3}, & 1\leq \left|w\right|<2,\\ 0, & \left|w\right|\geq 2. \end{array}\right. \end{array}$$

The calculation method of multisampling pooling is as follows:

(4) $$\begin{array}{c} f\left(i+u,j+v\right)=ABC, \end{array}$$

where *A*, *B*, and *C* all represent matrices and the specific form is as follows:

(5) $$\begin{array}{c} A=\left[\begin{array}{cccc} S\left(1+u\right) & S\left(u\right) & S\left(1-u\right) & S\left(2-u\right) \end{array}\right],\\ B=\left[\begin{array}{cccc} f\left(i-1,j-2\right) & f\left(i,j-1\right) & f\left(i+1,j-2\right) & f\left(i+2,j-2\right)\\ f\left(i-1,j-1\right) & f\left(i,j-1\right) & f\left(i+1,j-1\right) & f\left(i+2,j-1\right)\\ f\left(i-1,j\right) & f\left(i,j\right) & f\left(i+1,j\right) & f\left(i+2,j\right)\\ f\left(i-1,j+1\right) & f\left(i,j-1\right) & f\left(i+1,j-1\right) & f\left(i+2,j+1\right) \end{array}\right],\\ C=\left[\begin{array}{cccc} S\left(1+v\right) & S\left(v\right) & S\left(1-v\right) & S\left(2-v\right) \end{array}\right], \end{array}$$

where *f*(*i*, *j*) represents the pixel value at the pixel point *t*(*i*, *j*), *f*(*i*+*u*, *j*+*v*) represents the pixel coordinates in the original image, and *u* and *v* represent the pixel coordinates in the both horizontal and vertical directions.

In the process of using MS-pooling operations, assume that the original image size is *m* × *m* and the image size after using MS-pooling is *M* × *M*. According to the ratio between the original image and MS-pooling image, the final coordinates of the image *F*(*X*, *Y*) are determined by the following formula:

(6) $$\begin{array}{c} O\left(x,y\right)=O\left(X*\frac{M}{m},Y*\frac{M}{m}\right)=f\left(i+u,j+v\right). \end{array}$$

Convert it into a MS-pooling function, whose function form is as follows:

(7) $$\begin{array}{c} F=\text{interpolation\_pool}\left(O,\left[M,M\right]\right), \end{array}$$

where interpolation_pool represents the MS-pooling operation, *O* is the input of the MS-pooling operation, *F* is the output of the MS-pooling operation, and [*M*, *M*] represents the image size using the MS-pooling operation.

**Figure 7:**
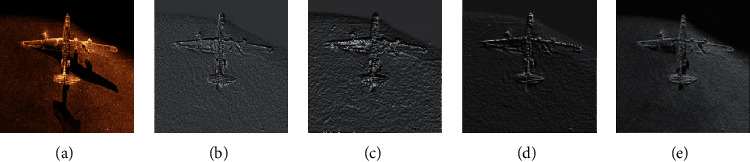
Different pooling operation results: (a) original image; (b) convolution feature; (c) mean-pooling; (d) max-pooling; (e) MS-pooling.

The pooling effect of different pooling operations is shown in Figure 7. The original image is first subjected to convolution operation to obtain a convolutional feature map, and then, a pooling feature map is obtained through pooling operation. Compared with the mean-pooling and the max-pooling, the MS-pooling maintains its original features as much as possible and can effectively avoid the transmission of its features in the multiple layer networks without causing feature loss and gradient disappearance.

### 3.3. QPSO Optimization Algorithm

The traditional CNNs model uses the backpropagation (BP) algorithm to complete weight parameter optimization. The BP algorithm tends to make the CNNs model training fall into local optimum, and the convergence speed is slow, which cannot guarantee the generalization ability of the network model. To solve the problem of BP algorithm, we propose the quantum-behaved particle swarm optimization (QPSO) algorithm to train the CNNs model. In the traditional particle swarm optimization (PSO) algorithm, the problem to be solved in optimization is to define a particle point in the *N*-dimensional search space. The particles fly in the search space at a certain speed and dynamically adjust the flight process according to their flight experience. In the process of flight search, each particle uses its current position and current speed, the distance between current position and particle best, and the distance between current position and global best to change its particle position. In the standard PSO algorithm, the particle swarm consists of *m* particles, and the position of each particle is assumed to be a possible preliminary solution to the problem in the *N*-dimensional search space. Particles update their flight trajectory based on their inertia, optimal position, and optimal group position.

**Figure 8:**
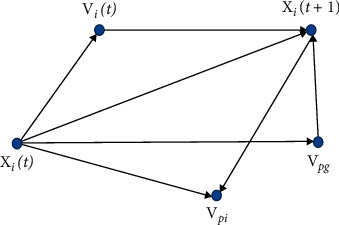
Diagram of particles moving in the PSO algorithm.

The idea of the PSO algorithm is to accelerate the close proximity of each particle to itself and the group. The moving process is shown in Figure 8. The starting position and velocity of the particle are randomly set in the solution space. During the iterative process, the PSO algorithm will record the optimal position experienced by individual particles and groups and the corresponding fitness function values. There are many problems in the traditional PSO algorithm, such as slow convergence and easiness to fall into local optimum and poor processing for discrete optimization problems. To solve the defects of PSO algorithm, we propose quantum-behaved particle swarm optimization (QPSO) algorithm. Based on the traditional PSO algorithm, we introduce the contraction-expansion factor and the differential evolution operator in the QPSO algorithm, which not only accelerates the optimization speed of the PSO algorithm but also prevents the algorithm from falling into the local optimal solution. The calculation formulas of the QPSO algorithm are as follows:

(8) $$\begin{array}{c} p_{i\;d}=\theta \cdot p{\text{best}}_{i\;d}+\left(1-\theta \right)\cdot g{\text{best}}_{d}, \end{array}$$

where *p*_*i* *d*_ represents the random position of particle *i* between *p*best_*i* *d*_ and *g*best_*d*_*A*.

(9) $$\begin{array}{c} m\text{best}=\frac{1}{M}\sum_{i=1}^{M}p{\text{best}}_{i}, \end{array}$$

where *m*best represents the center point of the current best position for all individuals.

(10) $$\begin{array}{c} k\left(t\right)=\frac{P_{g}\left(t\right)}{\text{avg}P_{i}\left(t\right)}, \end{array}$$

where *P*_*g*_(*t*) represents the optimal position of the population and avg*P*_*i*_(*t*) represents the optimal average position of the individual.

(11) $$\begin{array}{c} \alpha \left(t\right)=\left(\alpha_{o}-\alpha_{m}\right)\cdot \;\exp\left(-{\left(\frac{t}{T}\right)}^{2}\cdot k\left(t\right)\right)+\alpha_{m}, \end{array}$$

where *α*_*o*_ represents the initial value of the contraction-expansion factor, *α*_*m*_ represents the final value of the contraction-expansion factor, *t* represents the current number of iterations, and *T* represents the maximum number of iterations.

(12) $$\begin{array}{c} X_{i\;d}=\theta \cdot \delta \;d+\left(1-\theta \right)\cdot g{\text{best}}_{d}\pm \alpha \cdot \left|m{\text{best}}_{d}-x_{i\;d}\right|\cdot \;\ln\left(\frac{1}{u}\right), \end{array}$$

(13) $$\begin{array}{c} \delta =X_{K}-X_{j},\;i\neq j\neq k, \end{array}$$

where *X*_*i* *d*_ represents the position of the next generation particle *i*, *δ* represents the evolution operator, *g*best_*d*_ represents the global optimum of all particle positions in the current generation, *x*_*i* *d*_ represents the position of the current generation of the particle *i*, and *θ* and *u* represent the uniform distribution in the 0 to 1 between. Training the DS-CNNs using the QPSO algorithm requires calculating the position information of the particles according to equations (8) to (13) and updating the weights and thresholds of the network.

The training process of DS-CNNs based on the QPSO algorithm is as follows:

(1) Design the structure of the network model and initialize the weight parameters of the DS-CNNs.
(2) Initialize the position information of the particle swarm.
(3) Set the control shrink-expansion factor and initialize the *NU* ∈ (0,1).
(4) The average optimal position of the particle swarm is calculated according to equation (9).
(5) Randomly take values from 0 to 1 and determine if it is less than *NU* and update the particle position according to equations (11) and (12).
(6) The particles are mapped to the weight parameters of each layer according to the principle of mutual correspondence.
(7) Calculate the mean square error of the network and sort the particles according to their fitness. The mean square error function is calculated as follows:
  (14) $$\begin{array}{c} \text{Net}.E=\frac{1}{N}\overset{N}{\underset{i=1}{\sum }}\overset{H}{\underset{j=1}{\sum }}{\left(Y_{ji}^{D}-y_{ji}\right)}^{2}, \end{array}$$
where *N* represents the number of training samples input, *H*represents the number of neurons in the output layer, *Y*_*ji*_^*D*^ represents the expected output of the *j* output node of the first *i* sample, and *y*_*ji*_ represents the actual network output of the *j* node of the first sample.
(8) Update particle individual optimality and global optimality.
(9) Confirm whether the termination condition is met. If it is satisfied, exit the loop and output the global optimum, and use it as the weight parameter of the network. Otherwise, loop again to find the optimal solution.

### 3.4. Evaluation Indicator

In order to quantitatively analyze the segmentation accuracy of sonar images, the specificity (SP), sensitivity (SE), accuracy (Acc), Jaccard index (Jacc), and dice coefficient (dice) are used as evaluation criteria. SP represents the proportion of the negative values considered by the segmentation method and the values that are really negative in the ground truth. SE represents the proportion of the positive values considered by the segmentation method and the real positives values given by the ground truth. Acc represents how close the ground truth and the segmentation proposed by the methods are and the relation between their hits and errors. Jacc is a statistic metric that measures the similarity between the ground truth and the segmentation proposed by the methods. Dice is used to measure the similarity between two samples. The calculation formulas are as follows:

(15) $$\begin{array}{c} \text{SP}=\frac{N_{tn}}{N_{tn}+N_{fp}},\\ \text{SE}=\frac{N_{tp}}{N_{tp}+N_{fn}},\\ \text{Acc}=\frac{N_{tp}+N_{tn}}{N_{tp}+N_{fp}+N_{fn}+N_{tn}},\\ \text{Jacc}=\frac{N_{tp}}{N_{tp}+N_{fn}+N_{fp}},\\ \text{Dice}=\frac{2\cdot N_{tp}}{2\cdot N_{tp}+N_{fn}+N_{fp}}, \end{array}$$

where *N*_*tp*_ represents the number of true positive pixels and a true positive indicates that the pixel belongs to the objective area both in the predicted segmentation result and in the ground-truth segmentation result. The *N*_*fp*_ represents the number of false positive pixels, and a false positive indicates that the pixel is an objective area in ground-truth, but it is a background area in predicting the segmentation result. The *N*_*tn*_ represents the number of true negative pixel points. True negative indicates that the pixel is a background area in the ground-truth, and the predicted result is also a background area. The *N*_*fn*_ represents the number of false positive pixels, and a false positive indicates that the pixel is a background region in the ground-truth, but it is an objective region in the predicted segmentation result.

## 4. Experiment and Analysis

All experiments are conducted with Intel Xeon E5-2643v3@3.40 GHz CPU, 64 GB RAM, NVidia Quadro M4000 GPU, 8 GB of video memory, by CUDA Toolkit 9.0, CUDNN V7.0, Python 3.5.2, Tensorflow-GPU 1.8.0, Windows 7 64 bit operating system.

### 4.1. Benchmark Datasets

**Figure 9:**
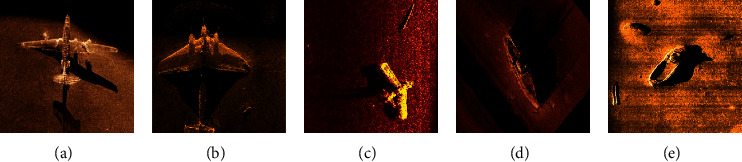
Original sonar image.

Sonar image dataset is collected by dual-frequency side-scan sonar (Shark-S450D), which is produced by Canadian Imagenex Company. The collection location is in Qingdao, China. Some original sonar images are shown in Figure 9. To use the sonar images dataset to train the weight parameters of the network model, the collected 3600 sonar images are proportionally divided into train dataset, verification dataset, and test dataset. We randomly select 60% of the dataset as training dataset, 30% of the sonar image dataset as verification dataset, and the other 10% as test dataset. Specifically, the train dataset includes 2160 sonar images, the verification dataset includes 1080 images, and the test dataset includes 360 images.

**Figure 10:**
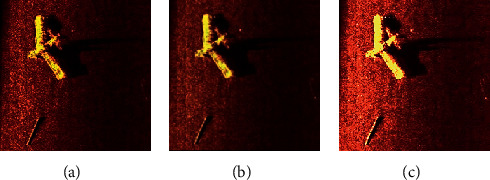
Sonar image preprocessing: (a) original image; (b) denoise; (c) enhance.

The average image size is 12000 × 8200 pixels, where the pixel values in the image are determined by the reflected strength of the echo and the distance from objects. Since the original sonar image contains much noise and is affected by the seabed environment and the sonar equipment, the collected images quality is poor. Therefore, the sonar image needs to be preprocessed. Firstly, the Gaussian filtering algorithm is used to denoise the image. Specifically, the sonar image is filtered using a 3 × 3 Gaussian filter template. Secondly, the linear interpolation algorithm is used to enhance the objective area in the sonar image. The preprocessed sonar images are shown in Figure 10.

**Figure 11:**
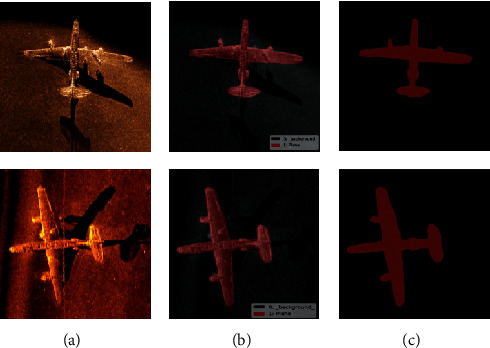
Original sonar images and corresponding labeled images: (a) original image; (b) manually label; (c) ground truth.

Since the original sonar images size is too large, it will occupy more GPU resources and increase the training time of the network models, and the original sonar image is reduced to 500 × 500 pixels. But directly reducing the size of the sonar image will result in loss of the information of the image. So we use a patch-wise method to solve this problem. As the ground truth for images is essential to use the supervised method for sonar image detection, we use the image annotation tool LabelMe, which is developed by the MIT to manually label images. The project source code is open. The original image and the corresponding manually annotated image are shown in Figure 11, where red represents the objective area and black represents the background area.

### 4.2. Network Model Training

**Figure 12:**
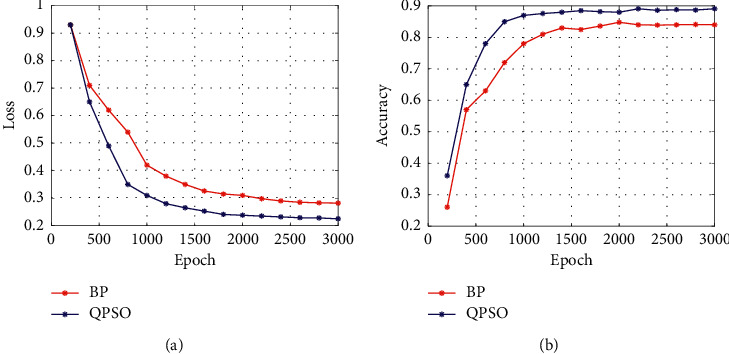
Training effect of different optimization algorithms.

In the training process of the network model, the BP algorithm is usually used to optimize the weight parameters of the network model, but in the DS-CNNs, the QPSO algorithm is used to optimize the weight parameters. In order to verify the superiority of QPSO algorithm in DS-CNN training, the BP algorithm and QPSO algorithm are used to train the network model in the sonar dataset. In the process of training, the batch size is set to 64, the number of epoch is 3000, and the accuracy is used as the segmentation evaluation standard. The training process and detection accuracy are shown in Figure 12.

It can be seen from Figure 12 that QPSO algorithm converges faster than BP algorithm in the training process. When the whole training is completed, the loss value of the QPSO algorithm is smaller than that of BP algorithm, indicating that the QPSO algorithm is suitable for training DS-CNNs. In the comparison of the segmentation accuracy results, the accuracy of QPSO algorithm is always higher than BP algorithm in the training process.

**Figure 13:**
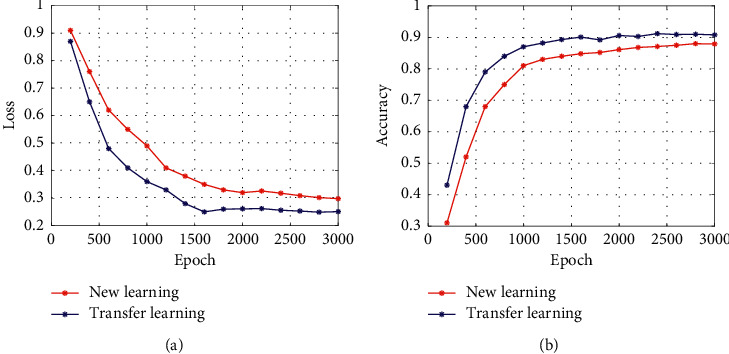
Comparison of different learning methods.

Due to the small size of the sonar dataset, the direct use of the network model training will lead to overfitting or low segmentation accuracy. The transfer learning is to transplant the model parameters trained in the large dataset to train ImageNet. In the network model, the training efficiency and robustness of the model can be improved. Therefore, in the training process of the DS-CNN model, the transfer learning method is introduced, and the network model parameters of the original U-Net network after repeated training in the large image dataset are used for the detection of sonar images. Common transfer learning methods include feature transfer and parameter transfer. Feature transfer is to eliminate the classification layer of the pretraining network and transform the feature map output from the convolution layer and the pooling layer into feature vector. And then, it sends feature vector to a new classifier for detection training. The parameter transfer is to randomly initialize the partial convolution layer and the remaining convolutional layers directly using the weight parameters of the pretrained network model and use the dataset to be trained to retrain the network parameters. In DS-CNN model, it is trained using a parameter transfer method for sonar detection. The initial learning rate of the network model is set to 0.001, the regular term coefficient is set to 0.001, and the number of epoch is set to 3000. The training effect of the two training methods of the new learning and transfer learning is compared in the sonar images dataset. The effect of the different training methods is shown in Figure 13.

It can be seen from Figure 13 that the use of transfer learning accelerates the convergence speed of the network model. As the transfer learning enhances the feature extraction capability, the network model achieves a higher detection accuracy.

### 4.3. Comparison of Different Convolution Kernels

**Figure 14:**
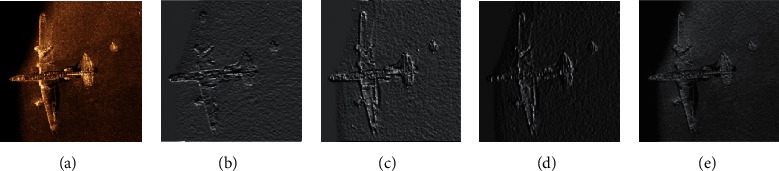
Feature extraction effect of different convolution kernels: (a) original image; (b) traditional; (c) dilated; (d) deformable; (e) dilated separable.

**Figure 15:**
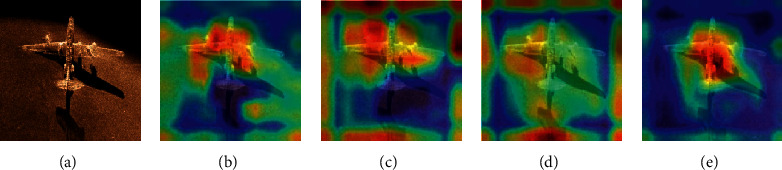
ROI of different convolution kernels: (a) original image; (b) traditional; (c) dilated; (d) deformable; (e) dilated separable.

In order to verify that the proposed dilated separable convolution kernel has a stronger feature extraction ability in sonar image and it is more suitable for sonar objective detective tasks, the traditional convolution, dilated convolution, deformable convolution, and dilated separable are used to compare the feature extraction effects of the sonar image. The feature extraction visualization results are shown in Figure 14. Since different convolution kernels have different regions of interest (ROIs) in the image, the heat map is used to visualize the convolution kernel feature extraction region. The heat map of the different convolution kernels is shown in Figure 15.

**Figure 16:**
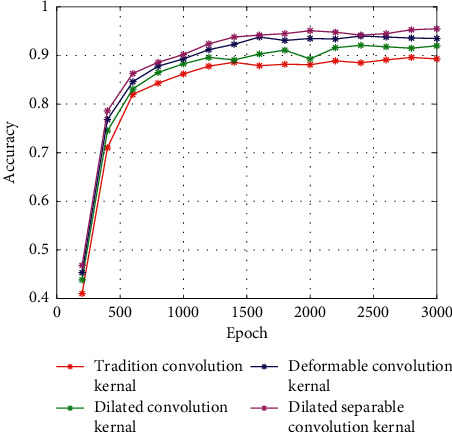
Detection accuracy of different convolution kernels.

It can be seen from Figure 14 the traditional convolution kernel has a poor feature extraction effect on the sonar image and a large amount of detailed image features are lost, resulting in an inconspicuous outline of the objective area. Since the dilated convolution uses different dilated coefficients to enlarge the local receptive field of the convolution kernel and enhance the feature extraction ability of the convolution kernel, it can extract the contour boundary of the objective, but the extracted boundary is fuzzy, and many related information is lost. Deformable convolution is poorly effective in feature extraction of sonar images. Since it is affected by noise in the sonar image, the deformable convolutions cannot completely extract feature for contour boundary feature extraction and detail feature extraction. The dilated separable convolution kernel not only extracts the contour features of the sonar image objective area but also enhances the ability to extract the detailed features by using the separable convolution, so it is suitable for the feature extraction task of the sonar images. It can be seen from Figure 15 that the ROI of the dilated separable convolution is concentrated, indicating that the dilated separable convolution has a better feature extraction effect on the objective area of the sonar image. Since different convolution kernels have an impact on the detection accuracy of sonar images, in order to verify the effectiveness of the dilated separable convolution for sonar image detection, we use traditional convolution kernel, dilated convolution kernel, deformable convolution kernel, and dilated separable convolution kernel to perform detection experiment in a sonar image dataset. The accuracy of detection is shown in Figure 16.

It can be seen from Figure 16 that the dilated separable convolution has a higher detection accuracy, and because it uses a separable structure, the detection accuracy in the first 500 epoch is higher than that of other convolution kernels. After 3000 epochs are completed, the final detection accuracy reaches 95%.

### 4.4. Comparison of Different Detection Methods

**Figure 17:**
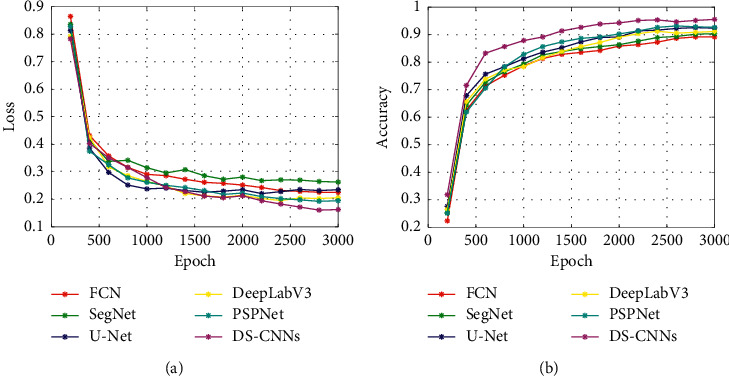
Training process and detection accuracy of different methods: (a) training process; (b) detection accuracy.

**Figure 18:**
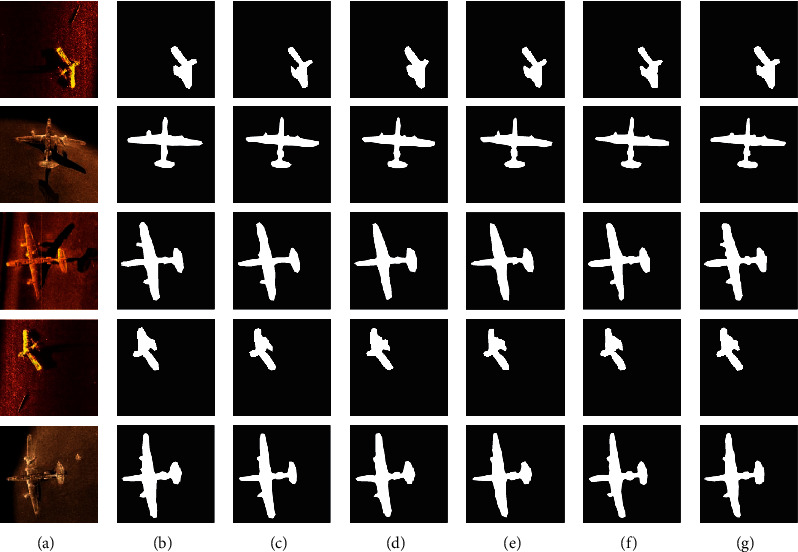
Sonar images detection results of different pixel sizes: (a) original; (b) FCN; (c) SegNet; (d) U-Net; (e) DeepLab; (f) PSPNet; (g) DS-CNNs.

In order to verify the effectiveness of DS-CNNs in sonar objective detection, DS-CNNs were compared with existing U-Net [38], FCN [39], SegNet [40], DeepLabV3 [43], and PSPNet [46] network models on sonar image datasets. The training process and detection accuracy of different methods are shown in Figure 17. The sonar objective detection experiment was performed on the sonar image dataset using DS-CNNs and other detection methods, and some of the visual results are shown in Figure 18.

It can be seen from Figure 17(a) that, during the training process, the DS-CNNs converge faster and the loss value is less fluctuating. FCN and SegNet converge slowly during training, and their loss value is still high when training is completed. U-Net converges faster during the initial training, but when it continues to train, the loss value gradually increases. The training process of DeepLabV3 and PSPNet is relatively stable, but the loss value is higher when the train is completed, which indicates that the training effect is poor under the same epoch times. The reason why the training effect is better is that it employs the dilated convolution kernel, which can reduce the parameter calculation of the network model and accelerates the convergence speed in the training process.  Figure 17(b) shows that DS-CNNs have a higher accuracy than the other methods in the detection of sonar objectives, indicating that DS-CNNs are suitable for sonar objectives detection tasks.

Figure 18 shows the results of different models on the sonar image test dataset. From the test results that the FCN, SegNet, and U-Net models can detect the contour region of the sonar objectives, the detection effect on the detailed information is poor, and there is information loss of the objective region. The DeepLab and PSPNet models have better segmentation of the detail feature of the objective region, but the effect is not good when detecting the background area. The results in Table 2 show that DS-CNNs have better effect in the sonar objective detection task in different evaluation indicators, and the average detection accuracy reaches 96.98%, indicating that DS-CNNs are suitable for sonar objective detection.

The calculation efficiency of different models is evaluated using the evaluation index of memory space, the number of parameters, training times, and test time. The calculation performance of different models is shown in Table 3. As can be seen from Table 3, DS-CNNs have the minimum values for different calculation efficiency evaluation indicators. The memory space during the training process is 3.42 GB, which ensures that DS-CNNs can be deployed on multiple mobile platforms. Moreover, the training time of DS-CNNs is 4.5 h, which indicates that the model is practical. The purpose of proposed DS-CNNs is to use it for underwater target detection. In the actual application process, underwater detection equipment must detect the location and contour of the underwater target timely and accurately, so the higher requirements are put forward for the real-time performance of the detection algorithm. The single image test time of DS-CNNs is 1.5 s, which can meet the real-time requirements of underwater objective detection.

### 4.5. Robust Analysis

**Figure 19:**
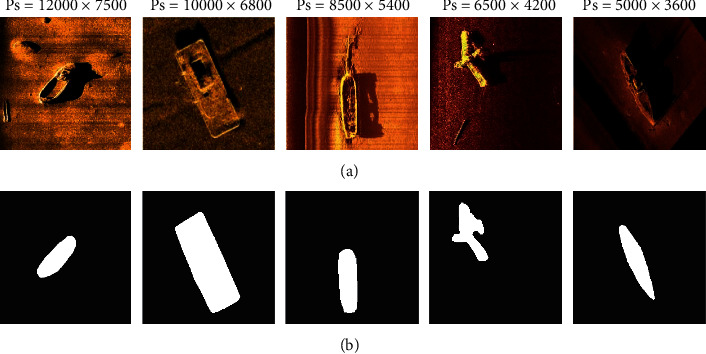
Sonar images detection results of different pixel sizes: (a) original sonar images of different pixel sizes; (b) detection results.

**Figure 20:**
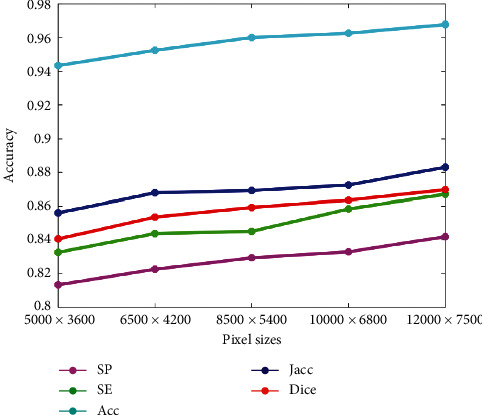
Detection accuracy of different pixel sizes.

Since the sonar image is taken underwater, the underwater complex environment will have an impact on the quality of the sonar image. Similarly, the quality of the sonar image also has an impact on the experiment results. In order to verify the effectiveness of the DS-CNNs, the proposed method is used to detect the sonar images of different pixel sizes (Ps), and the experiment results are shown in Figures 19 and 20. It can be seen from that as the pixel size increases, the detection accuracy of DS-CNNs increases gradually, which indicates that the pixel size has an impact on the detection accuracy, but the detection accuracy of DS-CNNs is around 95%, indicating that DS-CNNs are less affected by changes in pixel size.

**Figure 21:**
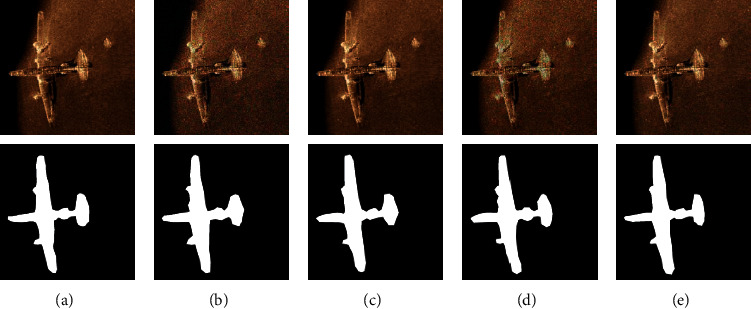
Detection results of different noise backgrounds: (a) original image; (b) Gaussian noise; (c) Poisson noise; (d) multiplicative noise; (e) salt noise.

Due to the influence of the seafloor environment, such as underwater acoustic channel, hydrological medium, and electromagnetic wave transmission, the imaging characteristics of sensor, high noise, and weak boundaries are commonly detected in the sensor images of detection region. The speckle noise has the most considerable influence on the detection of sonar image. In order to verify the detection accuracy of DS-CNNs in the noise background, the Gaussian noise, Poisson noise, multiplicative noise, and salt noise with a signal-to-noise ratio (SNR) of 35 are added to the original sonar image. The detection experiment of the sonar image with noise was performed using DS-CNNs, and the results are shown in Table 4 and Figure 21. It can be seen that the DS-CNNs still have a favorable detection effect on the sonar images under noise background, in which Poisson noise has the least influence on the detection effect and the average detection accuracy reaches 95.12%. Under the background of Gaussian noise, the detection effect is poor, but the average detection accuracy also reaches 93.28%. The detection results under the noise background show that DS-CNNs are robust to noises.

**Figure 22:**
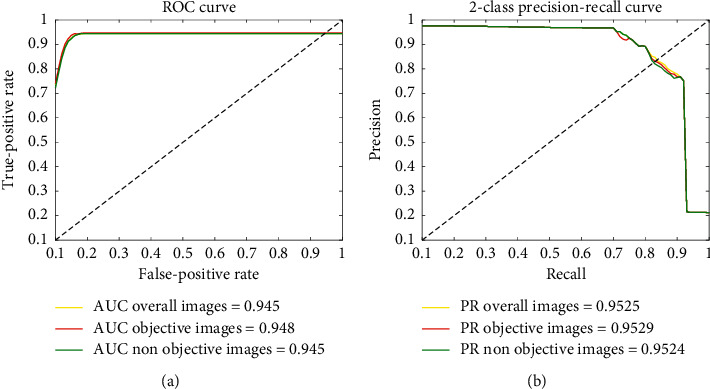
ROC and AUC of DS-CNNs.

Objective detection task is performed in randomly selected 1850 sonar images using DS-CNNs, and the 1850 sonar images include both objective and nonobjective types. The receiver operating characteristic (ROC) and area under curve (AUC) were used to evaluate the effect of the model on 1850 sonar images. The ROC and AUC of DS-CNNs are shown in Figure 22. It is analyzed from Figure 22 that the models have excellent stability in the sonar objective detection task. The detection accuracy of the sonar image with objective and nonobjective is more than 94.5%, indicating that the DS-CNNs are applicable to solve the problem of sonar objective detection.

## 5. Conclusion

Accurate detection of underwater sonar objective is an important task for underwater detection. In order to solve the shortcomings of the existing detection methods, this article proposed a novel network, DS-CNNs, for detection of underwater sonar objective. The innovations of DS-CNNs include the dilated separable convolution kernel, multisample pooling, and QPSO optimization algorithm. In the experiment process, it is found that the dilated separable convolution kernel can effectively extract the multiscale feature of the sonar objective; the multisampling pooling operation can reduce the loss of objective feature information; the QPSO optimization algorithm can speed up the model training speed and alleviate the overfitting phenomenon. To evaluate the effectiveness of the proposed method, DS-CNNs are used to perform objective detection experiments on sonar image datasets and compared with existing methods.

Experimental results show that the detection accuracy of DS-CNNs reaches 96.98%, which is better than other compared methods. Moreover, DS-CNNs have short model training time and few calculation parameters, so it has strong practicability. In the process of robustness evaluation, the proposed method still has high-objective detection accuracy for sonar images containing noise, indicating that DS-CNNs have strong robustness to noise and underwater environmental changes. In future work, we will concentrate on how to reduce its number of iterations and shorten its running time. In addition, we will investigate how to compress network model parameters and apply them to the practical application of underwater portable devices.

## Figures and Tables

**Table 1:** Specific parameters of DS-CNNs.

Layer	Type	Kernel	Channel
Layer_1	Conv	3 × 3	32
Layer_2	Conv	3 × 3	32
Layer_3	Conv	1 × 1	32
Layer_4	MS-pooling	2 × 2	64
Layer_5	Conv	3 × 3	64
Layer_6	Conv	3 × 3	64
Layer_7	Conv	1 × 1	64
Layer_8	MS-pooling	2 × 2	128
Layer_9	Conv	3 × 3	128
Layer_10	Conv	3 × 3	128
Layer_11	Conv	1 × 1	128
Layer_12	MS-pooling	2 × 2	256
Layer_13	Conv	3 × 3	256
Layer_14	Conv	3 × 3	256
Layer_15	Conv	1 × 1	256
Layer_16	MS-pooling	2 × 2	512
Layer_17	Conv	3 × 3	512
Layer_18	Conv	3 × 3	512
Layer_19	Conv	1 × 1	512
Layer_20	MS-pooling	2 × 2	1024
Layer_21	DC	—	—
Layer_22	Conv	3 × 3	512
Layer_23	Conv	3 × 3	512
Layer_24	MS-pooling	2 × 2	512
Layer_25	Upsampling	2 × 2	256
Layer_26	DC	—	—
Layer_27	Conv	3 × 3	256
Layer_28	MS-pooling	2 × 2	256
Layer_29	Upsampling	2 × 2	128
Layer_30	DC	—	—
Layer_31	Conv	3 × 3	128
Layer_32	MS-pooling	2 × 2	128
Layer_33	Upsampling	2 × 2	64
Layer_34	DC	—	—
Layer_35	Conv	3 × 3	64
Layer_36	MS-pooling	2 × 2	64
Layer_37	Upsampling	2 × 2	32
Layer_38	DC	—	—
Layer_39	Conv	1 × 1	32
Layer_40	SoftMax	2	2

**Table 2:** Detection accuracy of different models.

Model	SP (%)	SE (%)	Acc (%)	Jacc (%)	Dice (%)
FCN	78.61	81.38	89.62	87.32	85.86
SegNet	79.26	83.25	91.02	88.97	87.06
U-Net	81.38	85.93	92.28	90.03	88.65
DeepLab	80.26	84.38	91.92	89.37	87.84
PSPNet	83.39	87.65	94.18	92.25	90.06
DS-CNNs	85.89	89.37	96.98	94.48	92.89

**Table 3:** Calculation efficiency of different models.

Model	Memory space (GB)	Training time (h)	Parameters
FCN	8.45	12.6	63,254,618
SegNet	6.13	10.3	56,421,514
U-Net	5.42	8.6	48,163,254
DeepLab	7.13	7.3	52,618,315
PSPNet	4.26	7.8	45,126,257
DS-CNNs	3.42	4.5	35,183,326

**Table 4:** Detection accuracy of sonar image with different noise types.

Noise type	SP (%)	SE (%)	Acc (%)	Jacc (%)	Dice (%)
Gaussian noise	78.62	81.26	93.28	83.35	84.57
Poisson noise	81.23	83.68	95.12	86.01	87.32
Multiplicative noise	79.16	82.31	94.36	84.53	85.13
Salt noise	78.91	82.06	93.81	83.98	84.37

## Data Availability

The source code and sonar dataset used to support the findings of this study have been in the https://figshare.com/s/99620ba7a7bfff1400c2 repository.
